# Dr. Kinglake, in Reply to Mr. Wayte and Others, on Obstetric Practice

**Published:** 1816-07

**Authors:** 


*For the London Medical and Physical Journal.*

**Dr. Kinglake,** *in Reply to Mr. Wayte and Others, on Obstetric Practice.*

IT is much to be regretted that a philosophical subject, such as all inquiries connected with medical science ought to be regarded, cannot be discussed with becoming mildness and urbanity. It often happens that a zeal for the promotion of truth may not be sufficiently dispassionate to guard and measure its expressions in the most appropriate manner; but when force of language pourtrays a firm persuasion of correctness, it may be allowable: it strengthens the description intended to be given, and leaves nothing ambiguous or equivocal in the statement of opinion. This apology, however, is not applicable to those who descend from the legitimate ground of abstract argument, to deal in personal invective, to reproach motives, and condemn objects, instead of satisfactorily answering and refuting objections. Dr. Merriman has led the van of inapplicable and indecorous language in the controversy that has originated in some observations which I lately submitted to the public on the pernicious influence of indiscriminate man-midwifery. Ample experience had fully convinced me that my endeavours to correct what I regarded as licentiousness in obstetric practice was at once humane and vindicable. No personal invective, no intemperate expression, no criminal accusations, were authorised, or can at all avail in detaching me from a persuasion that rests on actual observation, not on verbose and groundless declamation.

Mr. Wayte, observing the dignified restraints of gentlemanly politeness in his first remarks on this subject, has been seduced from what appeared to be his better taste and judgment into an imitation of Dr. Merriman's illiberal asperity. Mr. Wayte, before he talks of my speculations exciting the "disgust of the whole medical world," should have reasoned much more conclusively and practically on the subject of his censure than he seems to be either willing or capable of doing.

Mr. Wayte, like Dr. Merriman and others, have, no doubt, their respective convictions on the subject, and they will as certainly retain them in opposition to my experience and reasoning; but they should keep their stand with a decorous and erudite liberality. Vehemence can never be substituted for argument, nor can personal abuse be merited even by erroneous reasoning. My opinions have been grossly mistaken, and as unjustly commented on. I contend only

 for for the *general* sufficiency of natural power in the parturine function, and fully admit the *occasional* necessity for the interference and aid of art. It is against the *ideal frequency* of unnatural occurrences, and the consequent occasion for uniform attendance, that I have unsparingly protested. I wish not to draw on myself the general opposition of obstetric practitioners, by any thing savouring of an attack on the particular judgment and conduct of individuals. It is no part of my object to be personal,—I have always deprecated such proceeding, as grovelling, and unworthy of scientific controversy. Dr. Merriman, Mr. Wayte, and others, unjustly feel indignant at my suspecting the correctness of the authorised practice of man-midwifery. On their side of the argument, it is not difficult to state cases, the circumstances of which would be imperative on the interference of the accoucheur. I will not, therefore, involve myself in a casuistic dispute, in which it would be almost impossible not to be bewildered without a chance of elucidating the doubt in question.

The occurrence of convulsions, uterine hæmorrhages, placental presentations, those of the face, mal-formation of the pelvis, disproportioned size of the fœtal head to the dimensions of the pelvic cavity, rigidity of the os uteri, and other possible instances, may happen, indispensably requiring obstetric aid. It is impossible to deny this obvious truth. It has never, indeed, entered into my imagination to question it; but I have and shall strenuously continue to withhold my assent to the assertion that these accidents are so *frequent* as to render uniform watching for their appearance absolutely requisite. This notion rests on a gratuitous assumption of the inadequacy of nature, *generally* speaking, to accomplish her destined function in the generative system of animal life. All my experience and inquiries on the subject fully satisfy me that the provisions of nature no more fail in insuring a due execution of the parturine office of life, than in performing any other vital function in the animal economy. The brain, the heart, the lungs, the stomach, the liver, &c. have their respective deviations from the natural standard, and their peculiar forms and characters of disease, so have the parturine actions of the uterus; but it cannot be justly contended that because nature is not uniformly perfect in sustaining the exigencies of animal health, that the diseased state is a legitimate order of things, and should be provided against as an inseparable and constant evil. This, indeed, would be to throw over the fair form of natural perfection the uncomely mantle of unsightly defect; it would be, in fact, mis-stating real circumstances, and substituting substituting the fallacies and incorrectnesses of human reasoning for the steady and salutary ordinances of natural provision.

Far be it from me to broach or countenance a doctrine that would diminish an *iota* of the care and attention due to impregnated females. They are, above all other claimants, entitled to the unremitted assiduities of a generous and feeling interest in their important situation. But their most needful and friendly aid on these occasions is unshaken confidence in the natural security in which they have been providently placed. No ground for apprehension can ever be reasonably entertained, from the general order of things, respecting their situation. If occasion for assistance should arise, it will be an *exception* to the ordinary course of experience, and, like all other exceptions to *general* rules, it may be remedied when it actually presents; but it can never be justly an object of fearful anticipation and of anxious care. Were human existence to be saddened by groundless dread that the *mere possibility* of an accidental organic derangement of health may be *incessantly* occurring, to what other end could the scheme of rational life have been instituted than to that of hopeless misery? Nature needs not my feeble advocacy to vindicate her from such inadmissible distrust of her competency to fulfil the high and gratifying destinations with which all her provisions are happily fraught.

I am not about to run the gauntlet of every obstetric attack that might be made on me from amongst the vast number of persons practising that art. No volume could be large enough to contain such instances as Mr. Wayte has cited in proof of what he holds to be necessary in midwifery practice. It would not become me to question, in the smallest degree, the correctness of that gentleman's narrative; nor could that liberty be taken with any of the immense mass of authorities which Dr. Merriman and Co. might adduce in support of their pre-conceived and determined views of obstetric practice. It would be also invidious in me to descend to instances calculated to rebut and annul the inferences that might be supposed to flow from such citations of urgent necessity; yet I will, in the simplicity and honesty of truth, state a few examples of cases of an opposite tendency to those of my opponent (Mr. Wayte).

Some years since, I was desired to visit, in consultation, a female suffering under what were regarded as ineffectual labour-pains. The surgeon who was in attendance, and who had assisted on three former occasions at the patient's accouchement, was well versed in the obstetric art, and was, in every respect, an intelligent, moral, and feeling man.

On

On seeing the patient, I was of opinion that the pains were preparatory only to eventual labour, and that, from their character and force, they were not likely to be efficient; that the patient had better be placed under the influence of thirty drops of laudanum, in the expectation that the real parturine action of the uterus would soon supervene. The object of my being consulted was to justify a practice that had been pursued in three former instances, by the same practitioner, in apparently similar circumstances, that of demolishing the fœtal head and delivering artificially. The practitioner referred to, with feelings of unfeigned humanity and known benevolence, that consecrated the purity of his intentions, seemed confident in the correctness of his opinion, that instrumental aid was indispensable, and strongly rested his persuasion on his experience on the three former occasions, in which, after waiting until the patient was enfeebled by protracted and unavailing pain, he was ultimately obliged to resort to that severe remedy. In the course of the consultation, I could not learn that any circumstances more than what existed at the time under consideration, were present in the former instances, so that the warranty of these former cases was equally afforded in that which formed the subject of consultation. The strong impression on the mind of the practitioner was, that the pains were inefficient, and that the strength of the patient would not admit of indefinite delay. The presentation was natural; no hæmorrhage, no convulsion; no fainting; the pulse was somewhat accelerated, but was firm and equal. The resolution was, however, at length taken to administer the opium, and to leave the patient to its soothing effect. The patient slept composedly during several hours after taking it, and then was awakened by increased pain, which soon became frequent, strong, and lasting, insomuch as to expel a full-sized healthy female child, with its appending placenta, without the smallest adventitious or personal aid. Indeed the event occurred before either the surgeon or myself, who were both called, could be in attendance. The sequel of the case was that of natural calmness and ultimate welfare both to the mother and the child. I state this case with undiminished respect for the professional talents and moral integrity of the practitioner concerned, who, instead of being at all disconcerted at the event, candidly and honestly acknowledged the important advantage that had resulted from the consultation.

Another instance of natural presentation recently occurred in which the state of apparent general exhaustion, inferred from the unavailing continuance of labour-pains during upwards wards of forty-eight hours, became an authority for destroying the fœtal head for the purpose of effecting delivery. The surgeon connected with this case is intelligent, humane, and well-intentioned. I had some conversation with him after the event, concerning it, and found him full of candour and honest feeling, not indignant at being questioned, but open to conviction, and discovering an exemplary disposition to profit by experience, and by a patient reliance, in similar circumstances, on the resources and capabilities of nature.

Some years have now elapsed since I was desired to see a female, at the full period of pregnancy, labouring under considerable uterine irritation, which had produced a dilatation of the os uteri of the dimensions of half-a-crown piece; the pains recurred at intervals of about five minutes, inducing increased arterial action, and a most harassing state of painful micturition. These symptoms proceeded but with little variation for several days and nights. Opium, in considerable doses, was given according to circumstances, which induced irregular sleep, from which the patient was always awakened by pain, exclaiming for "help, or she should die." This state continued ten days, no authority having been afforded, in my judgment, for instrumental aid. At that time, the deservedly eminent Dr. Clarke's opinion, of London, was solicited by letter, which he promptly gave by justifying the delay, adding, "that the patient was in safe hands that had so judiciously managed the case; and that he expected the event would be natural delivery." On or about the fourteenth day from the commencement of these pains, the real parturine action of the uterus took place, and the patient was speedily delivered of an healthy child. No untoward symptom ensued, and both the mother and her offspring did perfectly well. This case strongly shews that the provisions of nature are, generally speaking, fully adequate to eventual delivery; and that the hand of art should not be too officious and precise in determining the period when it should be accomplished. The time that might be the natural one in some cases, might be very unseasonable in others; and unless circumstances of imperious necessity should require the interference of art, nature should be left to work her own unperverted course, which, in general, in an immense majority of instances, will be found fully capable of executing most salutarily the function of parturition.

Numerous other instances, resembling the foregoing, might also here be stated, if it could be imagined that a reasonable objection could exist against the comments that I have offered fered on the too frequent practice of resorting to instrumental delivery in cases of natural presentation, unaccompanied by any of the indisputable warranties for that mode of assistance. I only require that natural power should be left at full liberty, that its energies should neither be thwarted nor unseasonably assisted, but that ample time should be allowed for a complete and undisturbed exertion of spontaneous effort, in the reasonable presumption that it will be ultimately found sufficient for all the purposes of safe, effectual, and timely delivery.

With this compromise with my opponents on the prevailing extent and mode of obstetric practice, I shall feel abundantly satisfied. It was never my design to effect any other change than that of conceding the point that natural presentations, unattended with either hæmorrhage, convulsions, or faintings, should not be considered as proper objects of instrumental aid, or of artificial delivery. With this admission the practice will always be safe; real occasions alone will demand the assistance of art, and, in these instances, it would be inhumanity and ignorance to withhold it. If my views are correct of the sufficiency of natural provision for its destined object, the solicitude which is naturally felt for the advantage and safety of obstetric practice will be allayed by the assurance that no interference will be unnecessarily made, and that such necessity will be indeed a very rare occurrence. On the vast scale of female impregnation always existing in every country, deviations from the natural order of things must now and then present, like all other exceptions to general rules; but then the exception cannot be made a basis on which to raise an argument for a frequency approaching even to general expectation, for the purpose of authorising an universal and an indiscriminate obstetric practice. If caution be carried the extreme length of attending in every instance of parturition, lest the rare accident of an exception to the general regularity and competency of nature should occur, I will not quarrel with such assiduous vigilance, provided the practitioner should not be betrayed into a disposition to lend assistance where none is really required, by considering that the efforts of nature are too tardy for his notion of artificial expedition. The popular delusion that something is necessary to be accomplished by the attendance of the accoucheur is mutually embarrassing to the patient and practitioner; and it must be allowed to be an awkward appendage, and to present a strong objection to the uniform practice of man-midwifery.

I shall now respectfully take my leave of the general obstetric practitioner on this subject, with an assurance that I have have not meant to give any individual the smallest offence, nor to have inculpated any one for mal-practice even, much less, as Dr. Merriman would have it, for "wilful murder!" The friends of liberal inquiry, and the experienced obstetric practitioner, will perceive the *quo animo* with which I have been actuated in the pending controversy, and will at once approve of my motive and object. With such persons I can have no dispute, no variance in moral estimation, nor shall I lose their future confidence in the justness of my intentions. But, against the speculative austerities of preceptors, the unrelenting irrascibility of disturbed prejudices, and the illiberal dogmatism of the unreflecting, I shall continue to wield the opposition of my own persuasion, and that of my unchangeable attachment to undissembled truth.

*Taunton;*
*May* 25, 1816.

